# The Efficacy and Safety of a Fixed Combination of Chinese Herbal Medicine in Chronic Urticaria: A Randomized, Double-Blind, Placebo-Controlled Pilot Study

**DOI:** 10.3389/fphar.2018.01474

**Published:** 2018-12-18

**Authors:** Sien-Hung Yang, Yi-Hsuan Lin, Jr-Rung Lin, Hsing-Yu Chen, Sindy Hu, Yi-Han Yang, Yi-Hsun Yang, Yin-Shuo Yang, Yao-Fan Fang

**Affiliations:** ^1^Division of Chinese Internal Medicine, Center for Traditional Chinese Medicine, Chang Gung Memorial Hospital, Taoyuan, Taiwan; ^2^School of Traditional Chinese Medicine, College of Medicine, Chang Gung University, Taoyuan, Taiwan; ^3^Chang Gung Immunology Consortium, Chang Gung Memorial Hospital and Chang Gung University, Gueishan, Taiwan; ^4^Graduate Institute of Clinical Medical Sciences, College of Medicine, Chang Gung University, Taoyuan, Taiwan; ^5^College of Medicine, Chang Gung University, Taoyuan, Taiwan; ^6^Clinical Informatics and Medical Statistics Research Center and Graduate Institute of Clinical Medicine, Chang Gung University, Taoyuan, Taiwan; ^7^Department of Dermatology, Chang Gung Memorial Hospital, Taoyuan, Taiwan; ^8^Department of Cosmetic Science, Chang Gung University of Science and Technology, Taoyuan, Taiwan; ^9^Royal Blackburn Hospital, Blackburn, United Kingdom; ^10^School of Dentistry, Matsumoto Dental University, Shiojiri, Japan; ^11^School of Medicine, Chung Shan Medical University, Taichung, Taiwan; ^12^Division of Rheumatology, Allergy, and Immunology, Chang Gung Memorial Hospital, Taoyuan, Taiwan

**Keywords:** chronic urticaria, Chinese herbal medicine, clinical trial, double-blind, placebo-control, randomization

## Abstract

**Background:** Chronic urticaria is a bothersome skin disease, and Chinese herbal medicine (CHM) is commonly used as adjuvant therapy. This study aimed to evaluate the effectiveness and safety of the mixture of two CHM formula, Xiao-Feng-San (XFS) and Qing-Shang-Fang-Feng-Tang (QSFFT), in treating urticaria through a randomized, double-blind, placebo-controlled clinical trial.

**Methods:** 78 participants entered the screening phase between November 2012 and August 2015. Participants were randomly and equally allocated in either CHM group (2 gm XFS and 2 gm QSFFT four times a day and 5 mg levocetirizine once daily for 28 days followed by 5 mg levocetirizine once daily alone for 28 days) or control group (placebo and 5 mg levocetirizine daily followed by 5 mg levocetirizine once daily for 28 days alone). Symptom improvement was set as the primary outcome, and the influence on sleep quality and changes in serum markers were used as secondary outcomes. Per protocol design was applied to the final analysis.

**Results:** A total of 56 participants entered the final analysis stage. Participants in the CHM group had more prominent symptom relief on day 56 (the weekly urticaria activity score, UAS7, as 9.9 ± 9.2 vs. 15.6 ± 10.8, *p* = 0.038). In the CHM group, participants' symptom severity reduced progressively (trend analysis, *p* < 0.001) while the decreasing trend was less favored in the control group (trend analysis, *p* = 0.056). The life quality improved gradually in both groups, while the differences between CHM and control groups were statistically insignificant. For urticaria-related cytokines, interferon-γ seemed to decrease positively in the CHM group (about 30.8% reduction from baseline, trend analysis *p* = 0.013). For safety issue, the CHM prescription was well-tolerated with no noticeable long-term side effects when compared to the control group. At 6-month follow-up of symptom changes after the end of the trial, the CHM group participants reported positive results in no recurrence or ≥50% improvement (36.3% in CHM group vs. 20% in Control group, *p* = 0.103).

**Conclusions:** The combination of XFS and QSFFT tended to be feasible and tolerable adjuvant therapy for urticaria in addition to standard therapy. However, larger study population with longer follow-up duration may be still needed.

**Trial registration:** NCT01715740 (ClinicalTrials.gov).

## Introduction

Urticaria is one of the most prevalent dermatological diseases, which is diagnosed with clinical manifestations of severe itching and recurrent wheals with central pallor (Bossi et al., 2011). The lifetime prevalence can be as high as 20% with any urticaria among general populations, and hence, it carries a heavy burden to the whole health system and patients' life (Ferrer, 2009; Zuberbier et al., 2009). The pathophysiology of urticaria is complicated, and thus, a long-term use of a combination of medications may be needed to control refractory symptoms (Fine and Bernstein, 2016; Kulthanan et al., 2016; Rimoldi et al., 2016). The candidate medications include non-sedating anti-histamines, glucocorticosteroids, cyclosporine, and leukotrienes antagonists (Cassano et al., 2006; Bossi et al., 2011; Takeda et al., 2011). Omalizumab, an anti-immunoglobulin E (IgE) antibody, is another potential therapy for urticaria, but its use is still limited because of high medication costs and possible symptom recurrence after discontinuation (Vichyanond, 2011; Maurer et al., 2013). Additionally, its side effects, such as systemic immunosuppression and endocrine disorders, and medication dependence remain as concerns to patients (Kaplan, 2012). Therefore, even under multiple Western medicine treatments, there are still unmet medical demands for symptom control in urticaria, and therefore, alternative treatments have been sought urgently (Magerl et al., 2010; Zazzali et al., 2012).

Accordingly, complementary and alternative medicine (CAM) becomes a possible option in addition to commonly used Western medication therapies. In the US, about 37% patients referred to the dermatology department of a tertiary care center have used at least one kind of herb supplement for their dermatologic problems (Kalaaji et al., 2012). In Taiwan, traditional Chinese medicine (TCM) is the most commonly used CAM, and Chinese herbal medicine (CHM) is the most commonly used modality of TCM. Other modalities include acupuncture, massage, and moxibustion (Chang et al., 2008; Chen et al., 2012). Among the more than 600 kinds of CHM used for urticaria in Taiwan, the core herbal formula is Xiao-Feng-San (XFS), which was identified by our previous published work based on the nationwide prescription database (Lin et al., 2013; Chen et al., 2015). XFS has anti-allergy, anti-oxidation, and anti-inflammation effects, and it can also recover the adequate balance between Th1 and Th2 immune systems (Akamatsu et al., 1998; Nose et al., 1999; Gao et al., 2005). About 50% of all prescriptions for urticaria contained XFS; however, the combinations of XFS with other CHMs with anti-inflammatory effects seem necessary in the whole CHM pharmacologic network for urticaria, in which nearly all CHMs used with XFS have an anti-inflammation effect (Chen et al., 2015). For this reason, the Qing-Shang-Fang-Feng-Tang (QSFFT), a famous CHM formula commonly used for inflammatory skin diseases, may become an ideal candidate medication for urticaria when combined with XFS. However, this combination is not as common as others and its effectiveness has not been proven yet (Chen et al., 2015, 2016).

This study aims to explore the efficacy and safety of combining XFS and QSFFT for treating urticaria as an adjuvant therapy to standard anti-histamine medication by conducting a randomized, double-blind, placebo-controlled clinical trial. Since CHM is often used as the complementary therapy in the clinical setting, the evaluation of symptoms and changes of serum markers are crucial for understanding the role of the fixed combination of XFS and QSFFT in urticaria. Moreover, the potential unwanted effects are also assessed for increasing concerns of CHM (Werner, 2014; Teschke and Eickhoff, 2015).

## Materials and Methods

### Study Design

**Figure 1:**
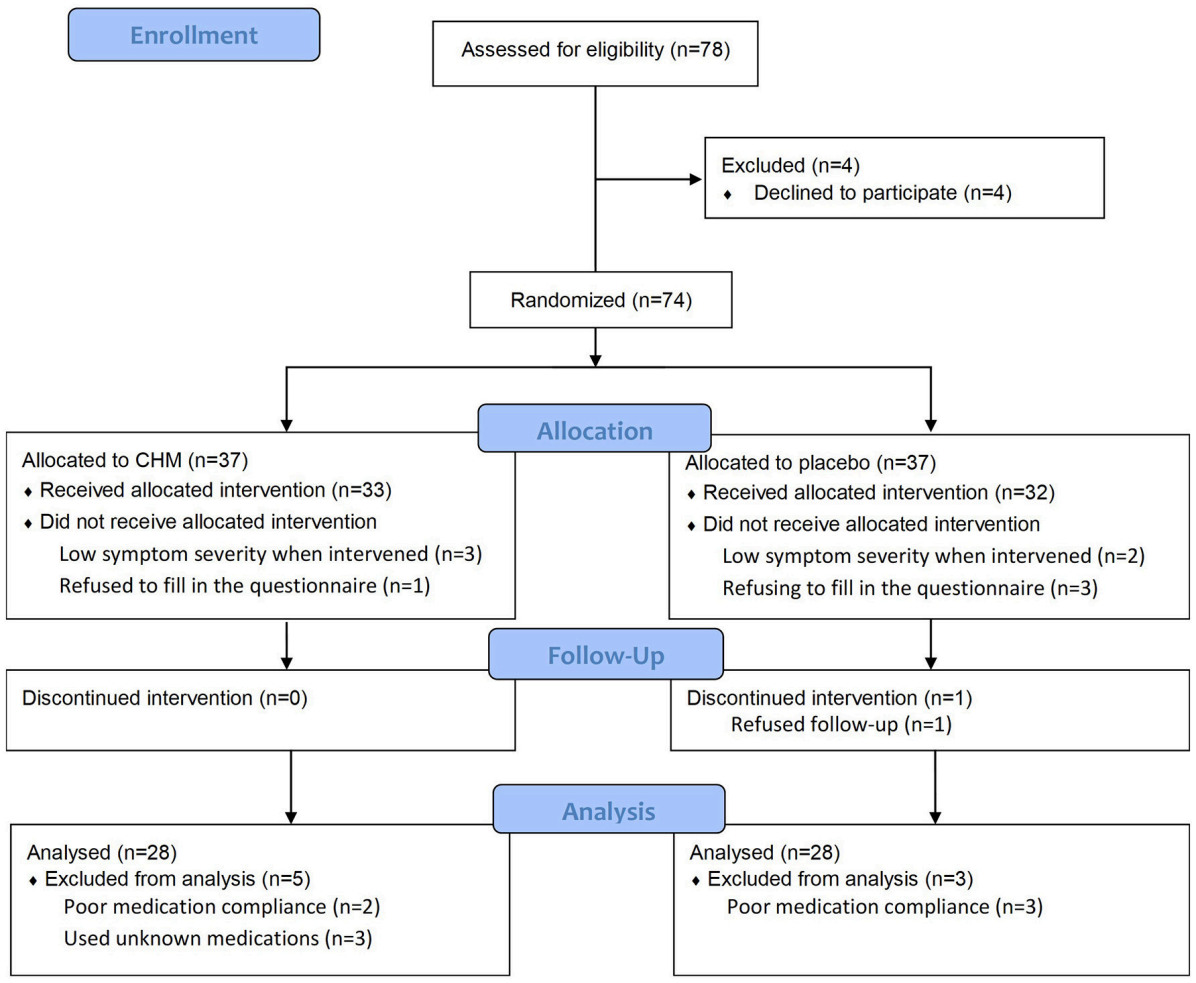
Flow diagram of this study (revised from the Consolidated Standards of Reporting Trials guideline). CHM, Chinese herbal medicine.

A two-arm randomized, placebo-controlled, double-blind clinical trial of 78 participants with urticaria was conducted at Chang-Gung Memorial Hospital (CGMH) in Taiwan (Figure 1). The subjects were recruited through advertisements and clinical visits and enrolled by dermatologists, rheumatologists, and TCM doctors, after considering the inclusion and exclusion criteria (Appendix A). All participants had written informed consent, and the trial was conducted in accordance with the Declaration of Helsinki. The study protocol was approved by the Institutional Review Board of the Chang-Gung Memorial Foundation (No. 101-2270A3) and carried out in accordance with the recommendation of the guideline for clinical trials of the same committee.

To closely resemble the clinical setting in the real world, in which combining CHM and Western medicine therapy is favored, we try to examine the benefit of add-on CHM therapy for urticaria. After screening for symptom severity and completeness of informed consent, all enrolled participants were given levocetirizine 5 mg once daily with either CHM or placebo medication for the first 4 weeks and then levocetirizine 5 mg alone once daily for the following 4 weeks. The first 4 weeks were referred to as the treatment phase (for short-term effect), while the following 4 weeks were the follow-up phase (for long-term effect). Levocetirizine was chosen as the comparative and baseline therapy in this study because of the recommendation from the dermatologist and rheumatologist in our research team. A total of three in-person visits was arranged for each participant for 8 weeks with the following schedule: day 0 (baseline), day 28 (end of CHM/control medication), and day 56 (end of trial). Two dates were also set to record symptom severity (days 7 and 35). Satisfaction surveillance through telephone contact was done for all participants 6 months after the trial finished. Outcome assessments were recorded using a self-assessed questionnaire, while blood samplings were done three times (days 0, 28, and 56) for urticaria-related cytokine assessment, differential blood count, and biochemical profiles of the renal and liver functions. Any adverse events, medication compliances, and concomitant other medications, including Western medicine, folk medicine, and herbal medicine, were recorded routinely by project staff. Baseline characteristic data, such as gender, birthday, comorbidities, food/drug allergy, and history of urticaria, were acquired at the first visit (day 0).

### Randomization and Allocation Concealment

Randomization was done by a trial administrator who was not involved in the clinical intervention or evaluation using a computer-generated random sequence before medications were administered. The random sequences evenly distributed all participants to two study arms. Medication bottles with opaque and identical appearance were filled by the medication manufacturer and were then labeled according to the generated random sequences. The pharmacy of CGMH was then provided with these medication bottles in consecutive number randomized as active and placebo. The enrolled participants were then given their interventions with the sequential number on the medication bottles. The randomization and allocation processes were adequately concealed and stored in a security box. All doctors, pharmacists, project managers, statisticians, and participants in this research were blinded to the allocation sequence throughout the entire trial phase.

### Chinese Herbal Medicine Formulation and Dosage

The CHM used in this study was a mixture of two well-known classical formulas, XFS and QSFFT, and each formula was produced at the same time by the Chuang Song Zong Pharmaceutical Company, which is a licensed good manufactured practice pharmaceutical factory in Kaohsiung, Taiwan. On the TCM's viewpoint, XFS is regarded as the important CHM formula to dispel exterior wind and heat, and this makes XFS become one of the most commonly used CHM for allergic skin diseases (Lin et al., 2013; Chen et al., 2015). On the other hand, QSFFT is used to enhance the efficacy of XFS on eliminating exterior heat. Both exterior heat and wind are thought to be the primary pathogenesis in urticaria. Wind represents that urticaria is a dermatologic (exterior) disease and the clinical course is often changing rapidly, while heat indicates the reddish and itching skin lesions of urticaria. For this reason, we combined XFS and QSFFT as the CHM treatment in this study as we treat urticaria patients in clinical practice. These two formulas were premade from raw ingredients according to the preparation methods recorded in the classics. In detail, an aqueous extract was obtained from the raw ingredients with a fixed proportion listed in Table 1, and the essential oil of QSFFT was recovered during the extraction process. Then, the concentrated powder of each formula was made separately by adding the extract to starch particles as excipients. No raw ingredients were added into the powder. Standard analytic methods were used to validate the composition of CHM (Appendix B), and the pesticide, microbiology contaminants, and heavy metal were all carefully examined according to the regulation in Taiwan. Similar to treating urticaria in daily clinical practice, we merely combined these two kinds of concentrated powder for the subjects in the CHM group. Additionally, the placebo was made from microcrystalline cellulose (49.7%), corn starch (49.7%), sodium carboxyl methylcellulose (0.6%), and food colorings (caramel, yellow #5, and yellow #6) to produce a similar taste, smell, and appearance of the pharmaceutically made CHM mixture. Both CHM and placebo were given in opaque capsules, weighing 500 mg per capsule. They were taken four times a day, eight capsules each time.

**Table 1:** The composition of Xiao-Feng-San (XFS) and Qing-Shang-Fang-Feng-Tang (QSFFT) used in the CHM group in this trial.

**XFS**			**QSFFT**		
**Name**	**Part used**	**w/w (%)**	**Name**	**Part used**	**w/w (%)**
*Saposhnikovia divariata (Turcz.) Schischk. (Fam. Apiaceae)* (Fang Feng)	Root	7.3	*Saposhnikovia divariata (Turcz.) Schischk. (Fam. Apiaceae)* (Fang Feng)	Root	10
*Atractylodes lancea (Thunb.) DC. (Fam. Asteraceae)* (Cang Zhu)	Dried rhizome	7.3	*Coptis chinensis Franch. (Fam. Ranunculaceae)* (Huang Lian)	Dried rhizome	5
*Schizonepeta tenuifolia (Benth.) Briq. (Fam. Laminaceae)* (Jing Jie)	Aerial part	7.3	*Ligusticum chuanxiong Hort. (Fam. Apiaceae)* (Chuan Xiong)	Dried rhizome	10
*Arctium lappa L. (Fam. Asteraceae)* (Niu Bang Zi)	Dried ripe seed	7.3	*Scutellaria baicalensis Georgi (Fam. Lamiaceae)* (Huang Qin)	Dried root	10
*Glycyrrhiza uralensis Fisch. (Fam. Fabaceae)* (Gan Cao)	Dried root or root with rhizome	3.7	*Platycodon grandiflorum (Jacq.) A. DC. (Fam. Campanulaceae)* (Jie Geng)	Dried root	5
*Rehmannia glutinosa (Gaertn.) Libosch. (Fam. Scrophulariaceae)* (Sheng Di Huang)	Dried root	7.3	*Forsythia suspensa (Thunb.) Vahl (Fam. Oleaceae)* (Lian Qiao)	Dried fruit	10
*Gypsum Fibrosum* (Shi Gao)	Mineral	7.3	*Mentha haplocalyx Briq. (Fam. Lamiaceae)* (Bo He)	Dried aerial part	5
*Caulis Clematidis armandii (Fam. Ranunculaceae)* (Mu Tong)	Dried stem	3.7	*Angelica dahurica Benth. et Hood. F. (Fam. Apiaceae)* (Bai Zhi)	Root	10
*Cryptotympana pustulata Fabricius (Fam. Cicadidae)* (Chan Tui)	Slough	7.3	*Schizonepeta tenuifolia (Benth.) Briq. (Fam. Laminaceae)* (Jing Jie)	Aerial part	5
*Sesamum indicum L. (Fam. Pedaliaceae)* (Hei Zhi Ma)	Ripe seed	7.3	*Citrus aurantium L. (Fam. Rutaceae)* (Zhi Qiao)	Dried immature fruit	5
*Sophora flavescens Ait. (Fam. Fabaceae)* (Ku Shen)	Dried root	7.3	*Glycyrrhiza uralensis Fisch. (Fam. Fabaceae)* (Gan Cao)	Dried root or root with rhizome	5
*Angelica sinensis (Oliv.) Diels. (Fam. Apiaceae)* (Dang Gui)	Dried root	7.3	Starch	Excipient	5
*Anemarrhena asphodeloides Bge. (Fam. Liliaceae) (Zhi Mu)*	Dried rhizome	7.3			
Starch	Excipient	13.8			

*The detailed information is also available online (https://dep.mohw.gov.tw/DOCMAP/lp-874-108.html)*.

### Outcome Assessment

#### Primary Outcome

The symptom severity of urticaria was designed as the primary outcome of this study and was obtained using self-administered, well-validated questionnaires, the weekly urticaria activity score (UAS7) and the Dermatology Life Quality Index (DLQI), on every visit (days 0, 7, 28, 35, and 56 for UAS7 and days 0, 28, and 56 for DLQI). Both questionnaires were commonly used for symptom assessment by clinicians and had been well-validated (Finlay and Khan, 1994; Khalil et al., 2015). These measurements were in high agreement with symptom severity and therefore were excellent to be used to assess symptom relief as the primary endpoint in this study.

#### Secondary Outcomes

Improvements in sleep disturbance were used as one of the secondary outcomes for patients with chronic urticaria since sleep disturbance is thought to be one of the commonest complications of urticaria in Taiwan, and one important ingredient of XFS is beneficial to insomnia (Yang et al., 2005; Lin et al., 2013). The validated Athens Insomnia Scale in Chinese version (CAIS-8), which is an eight-question questionnaire rated on a 0–3 scale, was used to evaluate insomnia with a great agreement with sleep disorders. People who score higher than six were considered to have sleep disorders (Soldatos et al., 2003; Chung et al., 2011). Furthermore, to understand the possible effects of the treatments on the immune system, examinations on urticaria-related cytokine changes in serum were studied, including IgE, eosinophil, C-reactive protein, interleukin (IL)-4, IL-6, IL-8, IL-10, IL-13, tumor necrosis factor-α, interferon-γ, and histamine. Also, trial medication compliances, other medications or healthy food intake, and concomitant other treatments for urticaria were required to be reported spontaneously, and all these events were recorded routinely during the whole trial period.

### Safety and Adverse Events Monitoring

All adverse events were requested to be reported to the project manager at any time they happened, and all suspicious events were closely monitored during the treatment and follow-up phases of the trial. The participants were encouraged to report any symptoms and discomforts, even unusual ones. Additionally, after giving proper information and explanation, blood tests were taken on days 0, 28, and 56. The complete blood count, aspartate aminotransferase, alanine aminotransferase, blood urea nitrogen, and creatinine were also checked before and after the trial was completed.

### Statistical Analysis

#### Sample Size Estimation

There are few clinical trials on the effects of CHM on urticaria. On the basis of one similar clinical trial, although the symptom severity measurement system was quite different, 41 participants per arm were needed to detect about 30% improvement in the active treatment group (Long et al., 2010). Under such circumstance, the 80% power at the significance of 0.05 can be achieved. For an expected dropout rate of <20%, a total of 100 enrollments in 1:1 ratio between the control and CHM arms were designed before the beginning of the clinical trial.

#### Statistical Methods

Counts and percentage were used to present categorical data, while mean with standard deviation to describe numerical data. To compare the initial status and outcomes between the CHM and control groups, χ^2^ statistics were used to examine the differences of categorical data, while independent *t*-tests to check the numerical data, including symptom scores and cytokine concentrations. Additionally, trend analysis for changes in symptom severity and cytokine concentration was carried out by one-way analysis of variances and paired *t*-test were used to examine the within-group differences before and after the CHM/placebo treatments. Finally, the self-report follow-up was conducted by phone contact at 6 months after the end of the trial. The symptom severity and recurrence were categorized as follows: no recurrence, ≥50% improvement, <50% improvement, and no improvement. In this case, χ2 statistics were used to examine the differences between the CHM and control groups. The sample size estimation before and after the study was carried out using G^*^Power (version 3.1), and the statistic tests were processed using SPSS (version 15.0, Chicago, IL, USA) with a per-protocol analysis. Any events that did not conform to the requirements of this trial design, such as missing data, poor medication compliance, and trial withdrawals, were reported in detail. In the case of missing values, the last value would be used. *P* < 0.05 or less than the *p*-value modified by the Bonferroni's method for multiple comparisons were thought to be statistically significant.

## Results

### Patients Recruitment

A total of 78 volunteer candidates underwent screening between November 2012 and August 2015, and four were excluded because they did not sign the informed consent and did not want to participate in the trial even after a detailed explanation. In August 2015, this trial was terminated because of fund shortage even though the preset goal of 100 participants was not achieved. Subsequently, a total of 74 patients underwent randomization and were allocated to either the CHM group or control group. Figure 1 shows the enrollment flow diagram in detail. In the treatment phase, levocetirizine with either CHM or placebo was taken from days 0 to 28. Four participants were excluded from the CHM group: three had too mild urticaria (with a UAS7 lower than 10 when the trial was initiated), and one refused to fill in the questionnaire. On the other hand, five participants were excluded from the control group: two only had a mild symptom, and three refused to fill in the questionnaire. Furthermore, in the follow-up phase, five participants were excluded from the CHM group (two had poor medication compliance, and three had unknown medication) and three participants from the control group (two for poor medication compliance and one quitted for no reason). Finally, the per-protocol analysis was done in 56 participants, with 28 members in each group, who completed the entire trial.

### Baseline Characteristics

The baseline characteristics of the CHM and control groups are summarized in Table 2. There were no statistical differences between the CHM and control groups, including patient age, gender, comorbidities, food/drug allergy history, previous duration of urticaria, past medication history for urticaria, initial UAS7, and biochemical profiles. Hemoglobin (Hb) levels between the CHM and control groups have a statistical difference, but both their Hb levels were within the normal range.

**Table 2:** Baseline characteristics of subjects (per protocol analysis).

	**CHM group *n* = 28**	**Control group *n* = 28**	***P*-value**
Gender			0.158
Male	21	16	
Female	7	12	
Age (years)	41.5 (14.09)	39.5 (13.92)	0.595
**COMORBIDITIES**			
Head and neck	2	3	1.000
Respiratory system	1	2	0.553
Cardiovascular system	2	1	0.553
Gastrointestinal system	1	0	0.313
Musculoskeletal system	0	3	0.075
Endocrine system	1	0	0.313
Genitourinary system	1	1	1.000
Neurologic system	0	1	0.313
Food/drug allergy	3	0	0.075
Previous duration of urticaria (months)	49.64 (69.06)	69.14 (83.49)	0.345
Past medication history for urticaria	11	7	0.146
**INITIAL PRESENTATION**			
UAS7	22.82 (9.29)	22.11 (8.17)	0.761
DLQI	8.54 (4.65)	9.39 (4.71)	0.496
CAIS8	6.50 (4.03)	7.04 (4.49)	0.641
**SEROLOGIC MARKERS**			
IgE (IU/mL)	241.75 (406.69)	449.10 (817.79)	0.235
Eosinophil (10^3^/μL)	0.26 (0.23)	0.20 (0.16)	0.327
WBC (10^3^/μL)	7.17 (2.30)	7.34 (2.17)	0.780
Hb (g/dL)	13.55 (1.39)	14.31 (1.25)	0.036^*^
AST (U/L)	22.93 (6.89)	22.39 (5.84)	0.755
ALT (U/L)	21.86 (13.98)	20.36 (10.24)	0.649
BUN (mg/dL)	12.43 (2.73)	12.98 (3.06)	0.481
Cr (mg/dL)	0.68 (0.18)	0.72 (0.18)	0.383
CRP (mg/L)	3.56 (8.16)	2.11 (3.50)	0.393

* *Statistics evaluated using the t-test for continuous data and X^2^ test for categorical data*.

*Age, previous duration of urticaria, scores of initial presentations, and levels of serologic markers were presented as mean (standard deviation)*.

*CHM, Chinese herbal medicine; UAS7, weekly urticaria activity score; DLQI, Dermatology Life Quality Index; CAIS8, Athens Insomnia Scale in the Chinese version; IgE, immunoglobulin E; WBC, white blood cell; Hb, hemoglobin; AST, aspartate aminotransferase; ALT, alanine aminotransferase; BUN, blood urea nitrogen; Cr, creatinine; CRP, C-reactive protein*.

### Primary Outcomes

**Figure 2:**
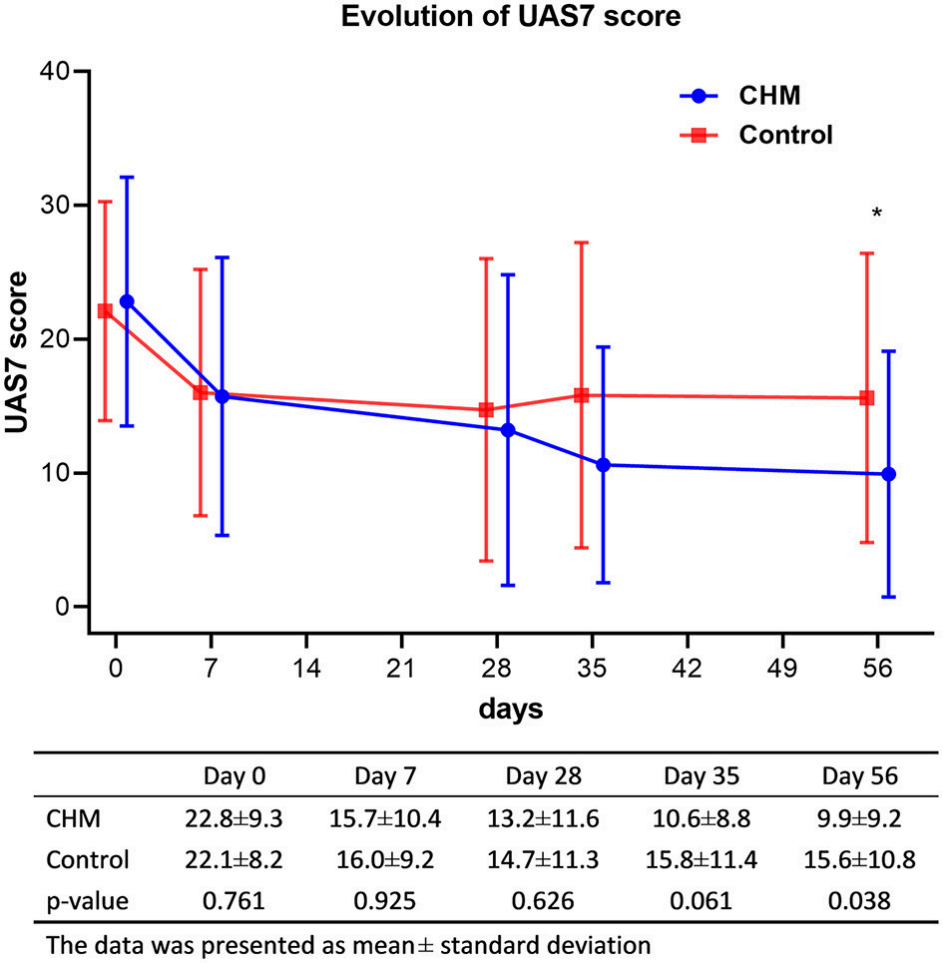
Evolution of symptom severity of urticaria (^*^p-value < 0.05 between the CHM and control groups). UAS7, weekly urticaria activity score; CHM, Chinese herbal medicine.

Figure 2 summarizes the changes in symptom severity of urticaria as the primary outcome. The trend analysis showed that symptom severity decreased progressively in the CHM group (*p* < 0.001) than in the control group (*p* = 0.056). An effective symptom relief could be found both in the CHM and control groups, and the symptom severity tended to be lower in the CHM group. The mean UAS7 in the CHM group was 22.80 at baseline and decreased to 13.2 at 28 days and 9.9 at 56 days. On the other hand, the mean score in the control group decreased from 22.1 at baseline to 14.7 at 28 days, and it slightly increased to 15.6 on 56 days. However, the differences in symptom severity between the CHM and control groups only achieved borderline significance when corrected by the Bonferroni's method (*p* = 0.038).

The improvements in life quality had a similar trend with that of the relief of symptom severity of urticaria. Nonetheless, both groups tended to improve gradually in life quality (both have *p* < 0.001 for trend analysis). On day 28, about 50% improvement can be observed in both groups, while the improvement became slightly better in the CHM group (Table 3). On day 56, DLQI continually decreased in the CHM group and slightly increased in the control group.

**Table 3:** Changes in the DLQI, sleep disturbance (CAIS8), and serum cytokines before (day 0), and after the treatment (day 28) and during the follow-up phase (day 56).

	**Day**	**CHM group**	**Control group**	***p*-value (CHM vs. control groups)**
DLQI	0	8.54 (4.65)	9.39 (4.71)	0.496
	28	4.89 (5.43)	4.54 (3.87)	0.778
	56	3.71 (4.13)	4.96 (3.97)	0.253
Trend analysis		*p* < 0.001	*p* < 0.001	
CAIS8	0	6.50 (4.03)	7.04 (4.49)	0.641
	28	5.46 (4.53)	5.07 (4.32)	0.745
	56	4.61 (4.43)	4.64 (3.47)	0.973
Trend analysis		*p =* 0.269	*p* = 0.072	
IgE (IU/mL)	0	241.75 (406.69)	449.10 (817.79)	0.235
	28	259.23 (467.90)	483.53 (920.46)	0.257
	56	253.50 (415.89)	432.36 (791.48)	0.295
Trend analysis		*p* = 0.988	*p* = 0.973	
Eosinophil (10^3^/μL)	0	0.26 (0.23)	0.20 (0.16)	0.327
	28	0.25 (0.24)	0.17 (0.12)	0.173
	56	0.20 (0.18)	0.20 (0.14)	0.993
Trend analysis		*p* = 0.547	*p* = 0.657	
CRP (mg/L)	0	3.56 (8.16)	2.11 (3.50)	0.393
	28	2.72 (5.49)	1.88 (3.22)	0.489
	56	2.26 (4.57)	1.89 (2.86)	0.724
Trend analysis		*p* = 0.734	*p* = 0.955	
IL-4 (pg/mL)	0	10.12 (4.81)	11.25 (5.43)	0.477
	28	10.25 (6.07)	10.32 (6.22)	0.973
	56	10.64 (5.89)	11.17 (4.34)	0.732
Trend analysis		*p* = 0.937	*p* = 0.774	
IL-6 (pg/mL)	0	22.01 (18.88)	42.54 (117.49)	0.495
	28	20.30 (9.76)	19.70 (12.39)	0.863
	56	22.58 (16.92)	28.12 (40.28)	0.568
Trend analysis		*p* = 0.852	*p* = 0.490	
IL-8 (pg/mL)	0	189.85 (288.77)	167.44 (228.13)	0.772
	28	245.25 (469.24)	103.85 (196.74)	0.137
	56	226.50 (360.85)	174.63 (340.11)	0.198
Trend analysis		*p* = 0.858	*p* = 0.542	
IL-10 (pg/mL)	0	9.71 (4.85)	17.89 (19.61)	0.060
	28	8.61 (4.27)	16.35 (17.82)	0.046^*^
	56	11.62 (10.72)	9.16 (8.62)	0.403
Trend analysis		*p* = 0.294	*p* = 0.102	
IL-13 (pg/mL)	0	4.26 (3.78)	3.39 (4.56)	0.503
	28	2.58 (2.35)	2.39 (3.34)	0.829
	56	4.31 (3.33)	2.30 (3.14)	0.047^*^
Trend analysis		*p* = 0.078	*p* = 0.483	
TNF-α (pg/mL)	0	10.43 (7.63)	12.27 (9.22)	0.484
	28	15.15 (6.37)	17.55 (19.92)	0.625
	56	13.07 (8.61)	14.29 (9.56)	0.662
Trend analysis		*p* = 0.072	*p* = 0.358	
IFN-γ (pg/mL)	0	42.72 (18.99)	39.65 (19.97)	0.901
	28	35.92 (10.58)	35.28 (22.17)	0.154
	56	29.56 (18.02)	41.85 (34.27)	0.105
Trend analysis		*p* = 0.013^*^	*p* = 0.636	
Histamine (ng/mL)	0	43.34 (18.21)	46.45 (19.46)	0.608
	28	47.57 (20.56)	48.71 (30.01)	0.884
	56	49.70 (26.45)	49.79 (18.92)	0.991
Trend analysis		*p* = 0.548	*p* = 0.862	

* *p < 0.05*.

*Data presented as mean (standard deviation)*.

*CHM, Chinese herbal medicine; DLQI, Dermatology Life Quality Index; CAIS8, Athens Insomnia Scale in the Chinese version; IgE, immunoglobulin E; CRP, C-reactive protein; IL, interleukin; TNF-α, tumor necrosis factor-α; IFN-γ, interferon-γ*.

### Secondary Outcomes

#### Sleep Disturbance

Table 3 shows that the improvements in sleep disturbances were not different between the CHM and control groups, and the trend analysis showed that both groups had no positive tendency to improve insomnia.

#### Urticaria-Related Cytokines

The changes in serum cytokines are summarized in Table 3. The changes in serum IL-13 in the CHM group were prominently different from the control group. Serum IL-13 in the CHM group declined from 4.26 pg/mL at baseline to 2.58 pg/mL at day 28 and returned to 4.31 pg/mL at day 56. In the control group, serum IL-13 continually decreased from 3.39 pg/mL at baseline to 2.39 pg/mL and 2.30 pg/mL at days 28 and 56, respectively (*p* = 0.047). Besides, the downward trend of serum IFN-γ could be seen in the CHM group, and the differences between days 0 and 56 were larger in the CHM group (42.72 and 29.56 pg/mL, respectively) than in the control group (39.65 and 41.85 pg/mL, respectively; trend analysis, *p* = 0.013).

### Safety Evaluation

All adverse events during the study period were reported and well-followed-up (Table 4). No significant difference in the incidence of adverse events between the CHM and control groups was seen (*p* = 0.252). Although the incidence seemed higher in the CHM group (21.4%), the causal relations were not definite. No deaths, anaphylactic shock episodes, or other major imbalances in any system organ affected by adverse events were reported during the study period. Adverse events reported included sweating, constipation, chest tightness, dizziness, herpes zoster, common cold, and sneeze. Additionally, the evaluations on the liver and kidney function profiles were all within the normal range before and after the trials (data not shown).

**Table 4:** No differences between the CHM and control groups in adverse drug reaction reported during the study period (*p* = 0.252 by χ^2^ statistics).

**Phase**	**CHM group** ***n*** **= 6**		**Control group** ***n*** **= 2**	
Treatment	Sweating	1		
	Constipation	1		
	Chest tightness	1		
Follow-up	Dizziness	1	Sneeze	1
	Herpes zoster	1	Common cold	1
	Common cold	1		

*CHM, Chinese herbal medicine*.

### Follow-up Satisfaction

As a subjective outcome, the 6-month follow-up satisfaction was also assessed by phone contact with 42 available participants: 22 in the CHM group and 20 in the control group. The higher 6-month response rate was found in the CHM group (36.4% vs. 20%) with “no recurrence” or “improvement ≥50%.” However, the difference between both groups had no statistical significance (*p* = 0.054; Table 5).

**Table 5:** Symptom severity of patients 6 months after the end of the trial (*p* = 0.103 by χ^2^ statistics).

	**CHM group *n* = 22**	**Control group *n* = 20**
No recurrence	4	2
Improvement ≥50%	4	2
Improvement < 50%	10	5
No improvement	4	11

*CHM, Chinese herbal medicine*.

## Discussion

This study disclosed the tendency of reducing symptom severity of chronic urticaria by using the CHM combination, XFS and QSFFT, with levocetirizine through a standard randomized, placebo-controlled, and double-blind clinical trial design. The key findings were that the CHM formula might reduce symptom severity during the treatment phase and the effect might expand through the follow-up phase. The decreasing trend in UAS7 implied that the symptoms of urticaria, including flare-up area, frequency, and itching severity, could be controlled in a better manner than levocetirizine alone. Additionally, the decreasing trend could be seen in the changes in the DLQI, in which the participants in both groups reported significant improvement during the treatment and follow-up phases, and the borderline differences between the CHM and control groups could also be seen on day 56. Both CHM add-on levocetirizine and levocetirizine alone can effectively relieve symptoms of urticaria after 1 month of treatment. However, the participants with CHM treatment have longer and better control of urticaria even though CHM was discontinued for 28 days. Furthermore, although we only had the subjective assessment on symptom severity 6 months after trial completion and the statistical significance was not achieved, there was a tendency that add-on CHM therapy may lead to better and long-term symptom relief. Using levocetirizine alone seemed to be only effective during the treatment phase. This may imply that the add-on CHM therapy has a higher cost-effectiveness value and different molecular pathways are influenced by CHM other than levocetirizine (Nettis et al., 2006).

This finding also implies that the use of CHM prescription may be helpful in reducing the use of levocetirizine since a 28-day duration of CHM and levocetirizine combination could improve the symptoms for 2 months or perhaps even 6 months. According to the present clinical consensus, patients' symptom severity determines the use of levocetirizine and the need for more advanced therapies, including corticosteroids, immunosuppressant, and monoclonal antibodies (Beck et al., 2017). Because of the more severe adverse effects and the higher costs of the advanced therapies, patients with chronic urticaria may be reluctant to receive those therapies even though the levocetirizine-alone therapy has its limitation, as the sustained symptoms were observed in our study during the second month. For this reason, the addictive effect of CHM combined with levocetirizine would become important for patients with chronic urticaria, so that a higher dose or longer duration of levocetirizine and the advanced therapies would not be necessary. However, a more extended treatment period and more study participants may be needed to make the differences more substantial.

Furthermore, the changes in serum immunologic markers may be helpful to recognize the potential mechanisms of CHM. INF-γ tended to decrease more prominently in the CHM group. INF-γ was reported as an indicator of autoreactive CD4^+^ T cells targeting FcεRIa and was found higher among patients with chronic urticaria (Ying et al., 2002; Auyeung et al., 2016). Although Th2 was thought to be predominant in causing urticaria, the suppression on INF-γ may also play an important role in maintaining the balance of the immune system of patients with urticaria. The possible reason for the add-on effects of CHM formula maybe related to the plausible effects of the ingredients in the CHM formula to the anti-histamine effect provided by levocetirizine (Nettis et al., 2006). Although the pharmacologic mechanisms on a human were not acknowledged, XFS, which is one of the CHM formulas used in this trial, has the effects of inhibiting histamine release from mast cells, reducing inflammatory response by inhibiting neutrophil function, scavenging free radicals, and decreasing IL-4 and INF-γ *in vivo* (Akamatsu et al., 1998; Nose et al., 1999; Gao et al., 2005). Furthermore, the ingredients of another CHM formula, QSFFT, were reported to have a strong anti-oxidative stress effect *in vivo*, and oxidative stress was proven to be one of the crucial factors of chronic urticaria (Sagdic et al., 2011; Dilek et al., 2016; Zhang et al., 2016). These “multiple herbs, multiple target” pharmacologic effects were characteristics of CHM treatments and can be the potential explanation about add-on effects for urticaria.

Additionally, the remaining effect of CHM for relieving urticaria after medication discontinuation may imply that the 4-week washout period for the CHM trials is not enough to eliminate the entire effect of CHM. Previous clinical trial designs revealed that a 4-week washout period was thought to be reasonable for CHM intervention (Fung et al., 1999; Huang et al., 2014); however, this has not been proven yet. In this trial, the remaining and even more remarkable effects of CHM can be seen 4 weeks after discontinuation. This may imply that the washout period should be longer than 1 month to evaluate the efficacy of CHM in a crossover design.

This study has some limitations. First, the participant enrollment was not as expected because of insufficient funding. Therefore, some of the statistical evaluation between the CHM and control groups achieved only borderline statistical significance, even the difference between the two groups seemed substantial in clinical perspectives. However, we believe that the statistical significance would be achieved with a larger participant number under the impression of the marked tendency for CHM to be a candidate adjuvant therapy for urticaria. As one of the most crucial implications for further clinical studies, the optimum subject number should be 74 for each arm (a total of 148), which was calculated by effect size 0.57 (from the differences on day 56) with an α error of 0.0125 (corrected for four comparisons) and β error of 0.8 in 1:1 treatment-control design. Second, the CHM used in this study was premade concentrated powder, and we used only four important compounds to examine the composition of CHM instead of authenticating all its herbal ingredients. Although we did not have detailed data for each herbal ingredient, the four compounds were good enough to determine the composition of a CHM formula according to the regulation for producing CHM in Taiwan. Also, since these two formulas were premade and were the same as those used in clinical practice, the repeatability of the study results could be retained. Third, since this trial was conducted in a single medical center in Taiwan, the pathogenesis or management of chronic urticaria may be different from Western countries. Therefore, the generalizability of using XFS and QSFFT for chronic urticaria may be limited in the Chinese population, and an examination of the external validity is still needed.

## Conclusions

In clinical settings, CHM is one of the most commonly used adjuvant therapies to Western medicine in Taiwan, and this trial provides the proof that CHM therapy with XFS and QSFFT could be a potential adjuvant therapy to levocetirizine to control urticaria in a longer and better manner. Besides, the safety of CHM was also proven to be similar to levocetirizine alone and is well-tolerated. This work warrants further larger-scale clinical trials and also bench studies on XFS and QSFFT as the core and effective CHM treatments for managing urticaria.

## Author Contributions

S-HY is the principal investigator of this project, and Y-HL cooperate with S-HY for study design, supervision on trial progress, and manuscript writing. J-RL and H-YC are both responsible for study design and statistics calculation. SH, Y-HaY, Y-HsY, Y-SY, and Y-FF are collaborators with this work for expert opinions and subject enrollment. All authors have read and approved the final version of the manuscript.

### Conflict of Interest Statement

The authors declare that the research was conducted in the absence of any commercial or financial relationships that could be construed as a potential conflict of interest.

## Abbreviations

CHM: Chinese herbal medicine
ALT: Alanine aminotransferase
AST: Aspartate aminotransferase
BUN: Blood urea nitrogen
CHM: Chinese herbal medicine
CAM: Complementary and alternative medicine
CRP: C-reactive protein
Cr: Creatinine
IgE: Immunoglobulin E
QSFFT: Qing-Shang-Fang-Feng-Tang
CAIS8: The Athens Insomnia Scale in the Chinese version
CGMH: Chang-Gung Memorial Hospital
DLQI: Dermatology Life Quality Index
UAS7: Weekly urticaria activity score
TCM: Traditional Chinese medicine
XFS: Xiao-Feng-San.
