# Supplementary material for: The Efficacy and Safety of a Fixed Combination of Chinese Herbal Medicine in Chronic Urticaria: A Randomized, Double-Blind, Placebo-Controlled Pilot Study
Source: Front Pharmacol. 2018 Dec 18;9:1474. doi: 10.3389/fphar.2018.01474 (PMC6305335; doi:10.3389/fphar.2018.01474)

Appendix A. Study inclusion and exclusion criteria

| Inclusion criteria | Exclusion criteria |
| --- | --- |
| Diagnosed as chronic idiopathic urticaria at least 6 weeks by a dermatologist or rheumatologist  Ages from 18 to 75 years  Symptom severity must be above 10 points (UAS7 scoring)  Willing to complete questionnaires and take medicine as scheduled in this study  Volunteer for study enrollment and sign informed consent | Other dermatological diseases related to skin pruritus, assessed by a clinician  Other specific types of urticaria including physical, cholinergic, and cold urticaria  Systemic diseases, such as cancer, renal diseases, liver diseases, autoimmune diseases, and acute infectious diseases, diagnosed by a clinician  Using oral/injected steroid, leukotriene inhibitors, immunosuppressant, or other Chinese herbal medicine for 1 month before enrollment  Abnormal differential blood count and liver or renal function tests in laboratory examination  Women who are pregnant or are planning to conceive |

Appendix B. Identification of (a) Xiao-Feng-San and (b) Qing-Shang-Fang-Feng-Tang by high-performance liquid chromatography. Four compounds were chosen to examine the composition of each formula.

(a)


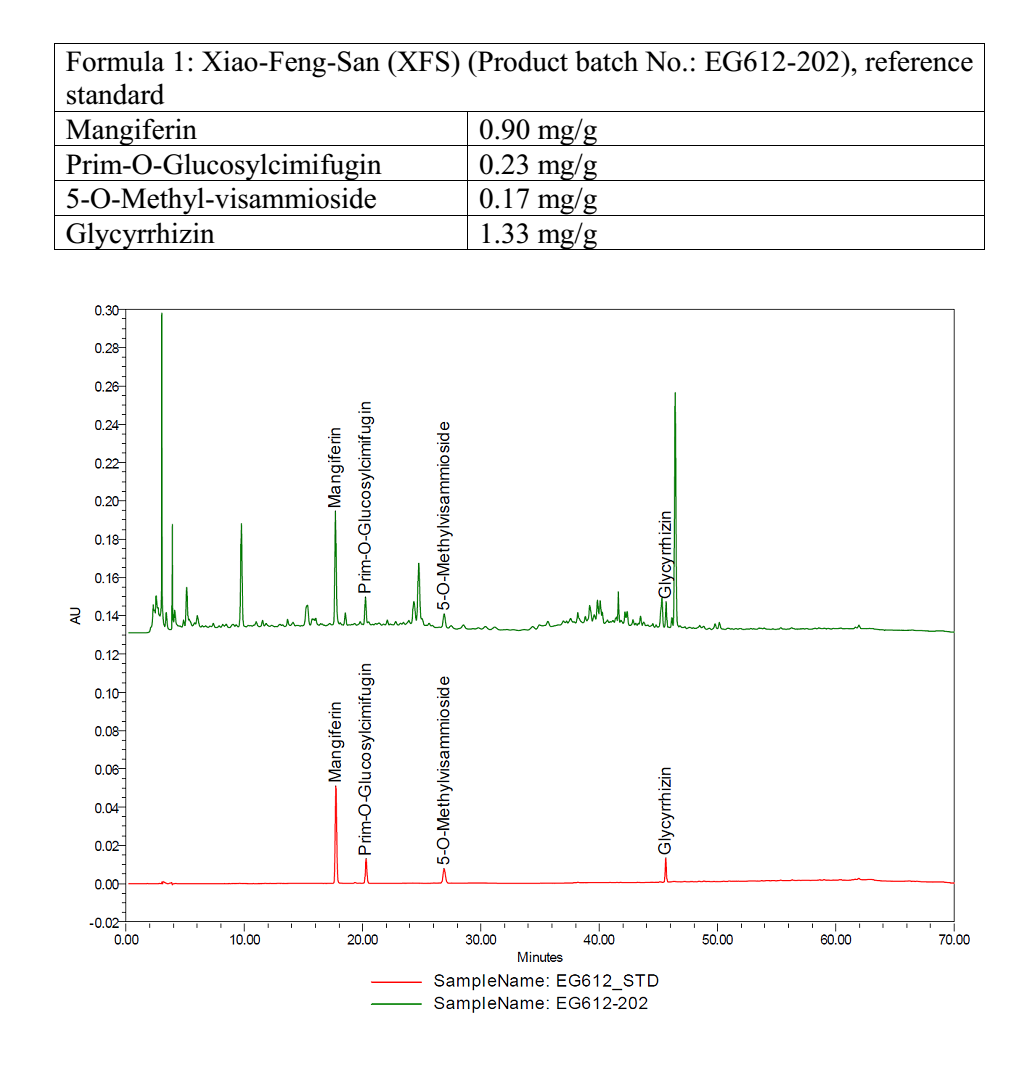


(b)


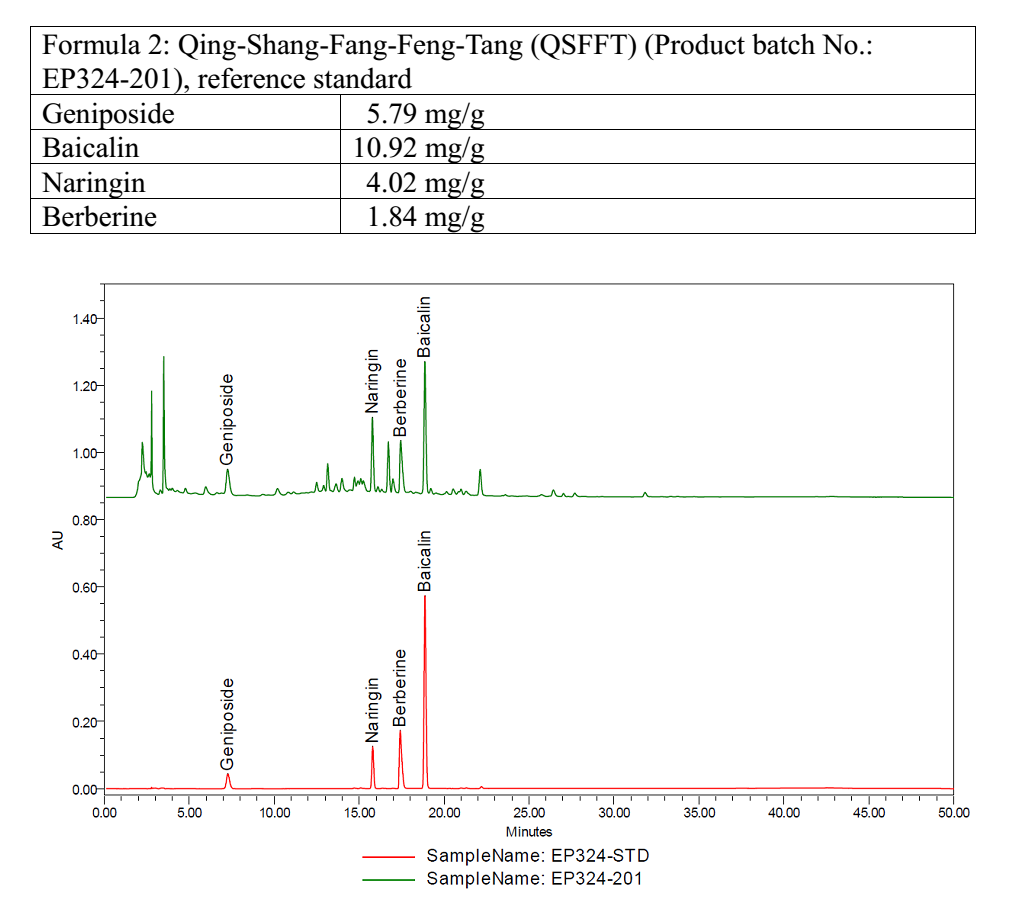

Supplement: Supplementary file 1 [file Table_1.docx]
